# Advanced Integration of Glutathione-Functionalized Optical Fiber SPR Sensor for Ultra-Sensitive Detection of Lead Ions

**DOI:** 10.3390/ma17010098

**Published:** 2023-12-24

**Authors:** Jiale Wang, Kunpeng Niu, Jianguo Hou, Ziyang Zhuang, Jiayi Zhu, Xinyue Jing, Ning Wang, Binyun Xia, Lei Lei

**Affiliations:** 1 National Engineering Research Center of Fiber Optic Sensing Technology and Networks, Wuhan University of Technology, Wuhan 430070, China; 317556@whut.edu.cn (J.W.); 318650@whut.edu.cn (K.N.); 318605@whut.edu.cn (J.H.); zzy03@whut.edu.cn (Z.Z.); zhujy@whut.edu.cn (J.Z.); 335936@whut.edu.cn (X.J.); 2 Zhongshan Institute of Modern Industrial Technology of SCUT, South China University of Technology, Zhongshan 528400, China; leilei@bacsense.com.cn

**Keywords:** optical fiber, surface plasmon resonance, glutathione, Pb^2+^ detection, gold nanoparticles

## Abstract

It is crucial to detect Pb^2+^ accurately and rapidly. This work proposes an ultra-sensitive optical fiber surface plasmon resonance (SPR) sensor functionalized with glutathione (GSH) for label-free detection of the ultra-low Pb^2+^ concentration, in which the refractive index (RI) sensitivity of the multimode-singlemode-multimode (MSM) hetero-core fiber is largely enhanced by the gold nanoparticles (AuNPs)/Au film coupling SPR effect. The GSH is modified on the fiber as the sensing probe to capture and identify Pb^2+^ specifically. Its working principle is that the Pb^2+^ chemically reacts with deprotonated carboxyl groups in GSH through ligand bonding, resulting in the formation of stable and specific chelates, inducing the variation of the local RI on the sensor surface, which in turn leads to the SPR wavelength shift in the transmission spectrum. Attributing to the AuNPs, both the Au substrates can be fully functionalized with the GSH molecules as the probes, which largely increases the number of active sites for Pb^2+^ trapping. Combined with the SPR effect, the sensor achieves a sensitivity of 2.32 × 10^11^ nm/M and a limit of detection (LOD) of 0.43 pM. It also demonstrates exceptional specificity, stability, and reproducibility, making it suitable for various applications in water pollution, biomedicine, and food safety.

## 1. Introduction

Lead pollution is abundant in the atmosphere, water, and soil, resulting in significant environmental pollution [1,2,3]. Additionally, it poses a threat to human health through the ecosystem, causing severe damage to the neurological, hematopoietic, and digestive systems [4,5,6]. The World Health Organization recommends that the lead content in drinking water should not exceed 10 ppb, and the U.S. Environmental Protection Agency sets the maximum allowable lead concentration in food at 72 nmol/L [7,8]. Therefore, there is an urgent need to develop effective technology for detecting lead ions (Pb^2+^).

Currently, the typical techniques employed for detecting Pb^2+^ mainly include fluorescence [9,10], electrochemical [11,12], colorimetric [13], and atomic absorption methods [14,15]. While these detection methods can effectively analyze the substances qualitatively and quantitatively, they suffer from limitations including low sensitivity, insufficient detection limitation, high detection cost, and complex operational procedures. Hence, it is highly significant to develop a Pb^2+^ detection method that possesses high sensitivity, and high accuracy as well as simple structure and low cost. In comparison to the aforementioned detection techniques, optical fiber sensing technology finds extensive application in areas such as temperature, strain, refractive index (RI), biosensing, and chemical sensing owing to its high sensitivity, immunity to electromagnetic interference, small size, low cost, ease of integration, and operational versatility [16,17,18].

Recently, a lot of optical fiber sensors have been designed and reported for Pb^2+^ detection, and by designing and optimizing both the micro-structure fiber and sensitive materials, optical fiber structures are primarily utilized to enhance sensor sensitivity. Additionally, the design of sensitive materials can further improve sensitivity while ensuring sensor specificity to the detector. Firstly, the reported microstructured optical fibers mainly include the Fiber Bragg grating (FBG) [19,20,21,22], D/U-type fiber [23,24,25], tapered fiber [26,27,28], de-cladding fiber [29], interferometric fiber [30,31], and end-reflective fiber [32]. These micro-structured fibers necessitate chemical or mechanical etching to remove the cladding, resulting in reduced durability and stability and complex manufacturing. Beyond that, their sensitivity is often required for further improvement. Recently, an optical fiber surface plasmon resonance (SPR) sensor utilizing multimode-singlemode-multimode (MSM) hetero-core fiber was reported. The sensor exhibits high sensitivity to changes in RI (3313.15 nm/RIU), along with excellent stability, durability, simple fabrication, and relatively low cost [33].

In addition, the selection of the appropriate sensitive material directly influences the detection accuracy of the sensor. Specific recognition and capture of Pb^2+^ is accomplished through the modification of various sensitive materials on the surface of the optical fiber. Several Pb^2+^-sensitive materials have been investigated, including chelating agents [34], molecularly imprinted polymers (MIPs) [35], nanomaterials [36], and biological materials [37]. Among these materials, chelating agents are commonly employed as sensitive materials for optical fiber Pb^2+^ sensors. They are favored due to their easy preparation and processing, low cost, and their ability to selectively form complexes with Pb^2+^, resulting in high sensitivity and selectivity compared to the other types of materials. However, certain chelating agents used in optical fiber Pb^2+^ sensors, such as Dimercaptosuccinic acid (DMSA) [38], Dimercaprol (BAL) [39], and Thioacetic acid (TAA) [40], are toxic, particularly to water or human samples, leading to contamination and potential harm. Glutathione (GSH) is a tripeptide compound consisting of glutamic acid, cysteine, and glycine. Both glutamic acid and cysteine contain carboxyl groups (-COOH), and the oxygen atoms within these groups can act as ligands, forming coordination bonds with Pb^2+^. The Pb^2+^ chemically binds to the deprotonated carboxyl groups in the amino acids through ligand bond formation, leading to the formation of stable and specific chelates [41]. Simultaneously, the three amino acids are linked together by peptide bonds, forming a linear GSH molecule. The linear structure of GSH provides it with a certain degree of flexibility and variability, enabling it to adapt to various spatial configurations and interactions, thereby specifically facilitating the formation of ligand bonds with lead ions. Furthermore, GSH is non-toxic, odorless, easy to prepare, and exhibits excellent biocompatibility. Consequently, combined with the high RI sensitivity of the SPR fiber, it is possible to develop various sensors with high sensitivity and excellent selectivity by different functionalization according to the corresponding detection targets.

In this work, an MSM hetero-core fiber modified with gold nanoparticles (AuNPs) and GSH is proposed for Pb^2+^ detection. Specifically, the sensing surface is formed by coating the single-mode fiber (SMF) with a layer of Au, onto which AuNPs and GSH molecules are self-assembled. The GSH molecule serves as a highly chelating sensitive material for Pb^2+^ detection. The modification of AuNPs enhances the SPR effect, thereby amplifying the optical sensing signal. A significant change in the SPR peak of the transmission spectrum occurs when there is a local RI change on the sensing surface of the SMF, caused by varying concentrations of Pb^2+^ in the medium being measured. The proposed sensor is characterized by a simple structure, easy fabrication, real-time monitoring, high sensitivity, low limit of detection (LOD), as well as good specificity, and excellent stability in the detection of Pb^2+^.

## 2. Working Principle

**Figure 1:**
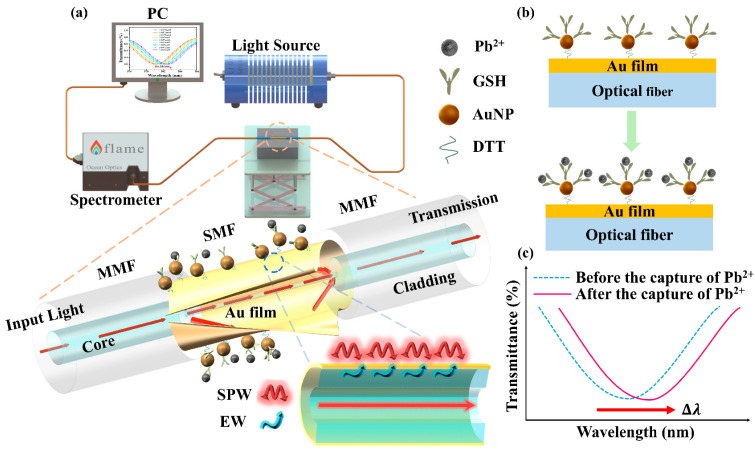
Schematic and working principle of the AuNPs/GSH-modified SPR optical fiber sensor. (**a**) Schematic of the AuNPs/GSH-modified SPR optical fiber sensor; (**b**) schematic structure of the sensing part of the AuNPs/GSH modified-SPR optical fiber sensor; (**c**) working principle of the AuNPs/GSH-modified SPR optical fiber sensor.

Figure 1a shows a schematic diagram of the structure and experimental setup of the proposed optical fiber Pb^2+^ sensor. The structure consists of a multimode fiber (MMF) as the signal transmission area and an SMF (15 mm) as the sensing area. The light signal emitted from the light source enters the core of the MMF and is transmitted into the SMF through total reflection in the core. Due to the large difference in diameter between the core of the SMF and the core of the MMF, the light signal emitted from the core of the MMF partially leaks into the cladding of the SMF, and total reflection occurs at the interface between the cladding of the SMF and the Au film and leads to the excitation of the evanescent wave (EW) [42]. The EW enters into the Au film and the AuNPs on the surface of the SMF, at the interface between the Au film and the medium to be measured, and the surface plasma wave (SPW) is excited, while at the interface between the AuNPs and the medium to be measured, the localized surface plasma wave (LSPW) is also excited. When the frequency of the EW matches that of the SPW and the LSPW, resonance occurs between photons and electrons, leading to the SPR effect and the localized surface plasmon resonance (LSPR) effect. The energy at the wavelengths corresponding to the EW is then coupled to the SPW and the LSPW, resulting in the attenuation of the reflected light signal. This signal is subsequently emitted from the core of the SMF. The output transmission spectrum can be recorded when the SPR/LSPR effect is excited on the fiber. Pb^2+^ in the medium to be measured forms specific and stable chelates with GSH on the surface of the optical fiber through ligand bonding (the specific binding mechanism of GSH to Pb^2+^ is shown in Figure 1b and Figure S1), which leads to a change in the local RI of the sensing region, causing a shift in the wavelength corresponding to the EW (Figure 1c). By detecting the one-to-one correspondence between the resonance wavelength and the Pb^2+^ concentration, the Pb^2+^ concentration can be determined. The LSPR effect induced by AuNPs also transfers a portion of the energy to the Au film on the optical fiber’s surface. This augmentation of the SPR effect on the Au film contributes to enhancing the sensitivity of the proposed sensor [43].

The principle of the Pb^2+^ detection sensor is summarized in Equation (1) [44]. In this equation, it is illustrated that when lead ions bind to the GSH located on the gold film surface, a small change in the GSH’s RI occurs. Consequently, the SPR effect is altered, leading to a shift in the position of the SPR resonance peaks. This systematic shift is then observed across the entire MSM spectrum. Ultimately, it manifests as changes in the wavelengths recorded in the monitored transmission spectrum.

(1) $$\Delta \lambda_{max}=m\Delta n\left\{1-\right.\exp(-\frac{2d}{l_{d}})\}\;$$

The amount of wavelength change ($\Delta \lambda_{max}$) in fiber optic SPR sensors can be calculated using the RI sensitivity (m), the change in RI (Δn) caused by the adsorbent material on the sensing region’s surface, the thickness of the adsorbent layer (*d*), and the length of electromagnetic field decay ($l_{d}$) on the optical fiber’s surface. Therefore, the concentration of Pb^2+^ solutions can be used as the detection target.

## 3. Material and Methods

### 3.1. Experimental Materials and Chemical Reagents

GSH was purchased from Macklin Biochemical Technology Co., Ltd. (Shanghai, China). DL-Dithiothreitol (DTT, 99%) was purchased from Aladdin Reagent Co., Ltd. (Shanghai, China). Phosphate buffered saline (PBS) was purchased from Sigma-Aldrich Trading Co., Ltd. (Shanghai, China). Sodium citrate (Na_3_C_6_H_5_O_7_.2H_2_O), chloroauric acid tetrahydrate (HAuCl_4_-4H_2_O), iron chloride (FeCl_3_), lead chloride (PbCl_2_), aluminum chloride (AlCl_3_), magnesium chloride (MgCl_2_), barium chloride (BaCl_2_), cobalt chloride (CoCl_2_), calcium chloride (CaCl_2_), sodium chloride (NaCl), and mercury chloride (HgCl_2_) were purchased from Sinopharm Chemical Reagent Co., Ltd. (Shanghai, China). SMF (core/cladding diameter, 8/125 μm) and MMF (core/cladding diameter, 50/125 μm) were purchased from Yangtze Optical Fiber and Cable Co., Ltd. (Wuhan, China). Deionized (DI) water was used to prepare the solution for this study.

### 3.2. Fabrication of AuNPs/GSH-Modified SPR Optical Fiber Sensor Probe

**Figure 2:**
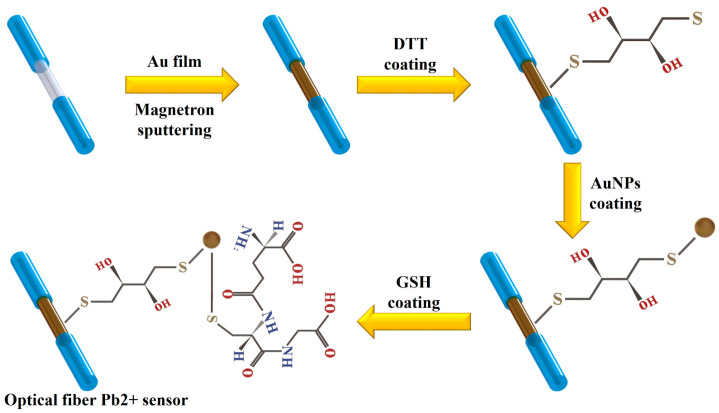
The processes to fabricate the AuNPs/GSH-modified SPR optical fiber sensor.

Figure 2 illustrates the fabrication process of a Pb^2+^ detection probe, which is modified with AuNP/GSH and consists of an MSM hetero-core fiber structure. In this study, the MSM hetero-core fiber structure is composed of a 15-mm-long SMF connected to two multimode fibers using a standard fiber fusion splicer and fiber cutter. To investigate the influence of SMF length on the sensing properties within the MSM heterocore structure, five distinct MSM architectures were fabricated, each with a different SMF length of 5 mm, 10 mm, 15 mm, 20 mm, and 25 mm. Following comprehensive analysis, it was determined that the optimal SMF length for the sensor was 15 mm (detailed results are presented in Figures S2 and S3, Tables S1 and S2). The fibers were subsequently washed multiple times with ethanol and DI water to eliminate the residual impurities and dust and then dried in a vacuum oven for further use. The sensing region of the optical fiber was sequentially coated with a 5 nm thick chromium layer followed by a 60 nm thick Au layer using a magnetron sputter coater. The MSM hetero-core fiber structure coated with a 60 nm thick Au film was subsequently fabricated.

To successfully modify the AuNPs on the surface of the sensor, the Au-coated optical fiber was immersed in a 1 mL DTT solution at room temperature for 1.5 h. DTT acts as a bridge, with one S-base bond of DTT connected to the Au film through an Au-S bond and the other S-base bond connected to the AuNPs through an Au-S bond. The DTT-modified optical fiber was then removed and rinsed multiple times with alcohol and DI water to eliminate any residual DTT that was not successfully modified on the surface. The successfully modified DTT optical fiber was then immersed continuously in the prepared AuNPs solution for 3 h while avoiding exposure to light. AuNPs were synthesized and their dimensional size (~20 nm) was controlled using a previously reported method (details of the preparation process can be found in Figure S4). The surface of the fiber was then modified with the synthesized AuNPs. Subsequently, the AuNPs-modified fiber was immersed in a GSH solution and shielded from light for 4 h. The S-base bond on GSH was used to bind with AuNPs through an Au-S bond. Finally, the optical fiber SPR sensor was successfully prepared with AuNPs/GSH modification on MSM optical fiber.

Additionally, we recorded the real-time stable transmittance spectra of the sensors after each material modification during the fabrication process. Figure S5 displays the transmittance spectra of the MSM optical fiber during the Au-film coating and modification with AuNPs and GSH. The transmittance spectra of the sensor, while modifying different materials on its surface, indicate noticeable wavelength drift. This observation confirms the successful modification of Au, AuNPs, and GSH on the surface of the optical fiber.

### 3.3. Instrument and Characterization

The MMF-SMF-MMF optical fiber was fabricated by using the fusion splicer (FSM-60s, Fujikura Ltd., Tokyo, Japan). The Au film was deposited onto the SMF section by magnetron sputtering (Bestec GmbH, Berlin, Germany). The absorption spectra of the sample solutions were characterized by the UV-Visible spectrophotometer (AvaSpec-ULS2048L, Avantes Co., Ltd., Beijing, China). The surface of the sensor and the AuNPs on the SMF were observed by scanning electron microscopy (SEM) (Zeiss G300, Carl Zeiss AG, Oberkochen, Baden-Wurttemberg, Germany). The elemental distribution on the surface of the fabricated sensor before and after the detection of Pb^2+^ was also analyzed by energy dispersive spectroscopy (EDS, Zeiss Sigma HD, Germany) and X-ray photoelectron spectrometer (XPS, Thermo ESCALAB 250Xi, Thermo Fisher Scientific Co., Waltham, MA, USA). Finally, the experimental setup included a halogen lamp light source (Ocean Optics HL-2000, Dunedin, FL, USA), an optical fiber spectrometer (Ocean Optics USB 2000+, Dunedin, FL, USA), and a light displacement stage.

### 3.4. Device Testing

Figure 1 shows the experimental setup used to study the performance of the sensor in detecting Pb^2+^. The sensor was placed in a V-shaped tank filled with a specific concentration of lead ions. One end of the sensor was connected to a halogen lamp light source, and the other end was connected to an optical fiber spectrometer. Different concentrations of Pb^2+^ solutions were prepared by diluting a 1 mM PbCl_2_ solution. The AuNPs/GSH-modified sensor was immersed in the different concentrations of Pb^2+^ solutions for 3 min. Once the spectra stabilized, the transmission spectra of the sensor were observed and recorded. Before each measurement, the sensing area of the sensor was washed with DI water and PBS solution to remove impurities and unreacted Pb^2+^. All experiments were conducted at a temperature of 25 ± 1 °C.

## 4. Results and Discussions

### 4.1. Characterization

To demonstrate the successful fabrication and effectiveness of the optical fiber sensor for detecting Pb^2+^, the materials modified on the fiber’s surface were characterized during its fabrication and Pb^2+^ detection.

**Figure 3:**
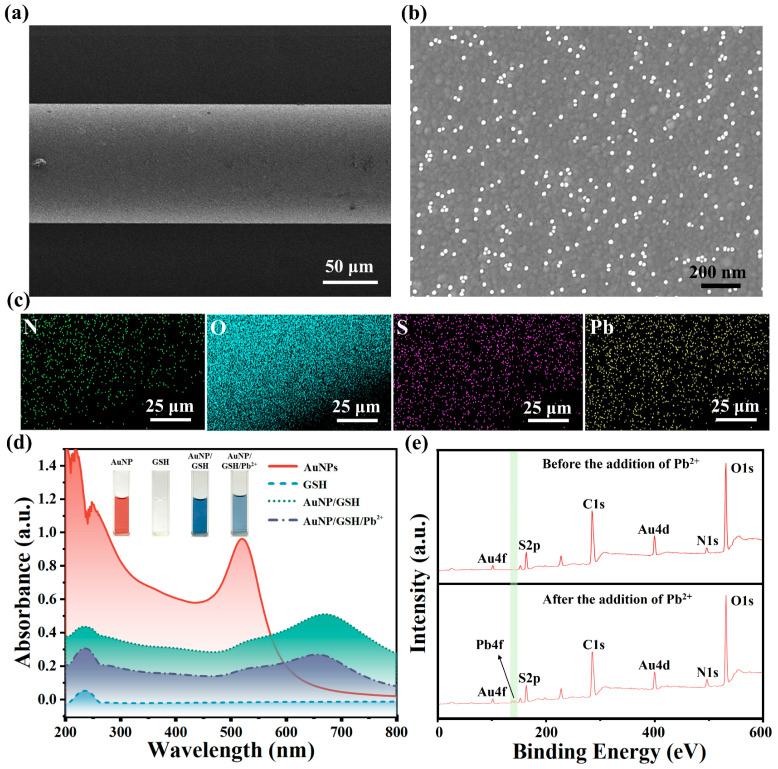
Characterization of the functional AuNPs/GSH on the fiber. (**a**) SEM characterization of the surface on the sensor; (**b**) SEM characterization of the fiber modified with AuNPs; (**c**) EDS analysis of the sensing probe surface after capturing Pb^2+^. (**d**) UV-vis of AuNPs, GSH, AuNPs/GSH, and AuNPs/GSH/Pb^2+^ and their separate solution states; (**e**) XPS survey spectra of the sensor obtained before and after Pb^2+^ detection.

The surface morphology and chemical composition of the AuNPs/GSH-functionalized fibers after Pb^2+^ capture were characterized using SEM and EDS, as depicted in Figure 3a–c. In Figure 3a, the SEM image displays a uniform and dense film on the surface of the AuNPs/GSH-functionalized fiber. Figure 3b shows an SEM image of the sensor surface after modification with AuNPs, revealing the uniform distribution of approximately 20 nm-sized AuNPs on the Au film surface. Figure 3c presents the elemental mapping analysis of N, O, S, and Pb on the optical fiber sensor after Pb^2+^ capture. The presence of S confirms the successful attachment of AuNPs/GSH to the fiber, while the presence of Pb demonstrates the sensor’s ability to detect Pb^2+^, illustrating the strong recognition and capture capabilities of GSH molecules towards Pb^2+^. Figure S6 displays the elemental distribution of the optical fiber sensor following Pb^2+^ capture, providing further evidence of the successful encapsulation of the AuNPs/GSH functional film on the surface of the fiber, enabling the detection of Pb^2+^ in aqueous solutions. Simultaneously, this sensor identifies Pb^2+^ by capturing it through surface-modified GSH. This process alters the RI within the sensing region, and these minute RI changes are converted into a detectable wavelength shift response.

The UV-visible absorption spectra of AuNPs, GSH, AuNPs/GSH, and AuNPs/GSH/Pb^2+^ during the fabrication and Pb^2+^ detection of the sensor are presented in Figure 3d. Among them, AuNPs exhibited an absorption peak at 520 nm, indicating their spherical shape [45]. The AuNPs exhibited a strong absorption peak at 520 nm, confirming their maximum absorption at this wavelength, while also displaying a secondary strong absorption peak at 234 nm. The self-assembled AuNPs/GSH exhibited a broad absorption peak at 671 nm, indicating the formation of Au-S bonds between AuNPs and GSH [46]. The addition of Pb^2+^ resulted in a decrease in the absorbance peak of AuNPs/GSH, providing evidence that the positively charged Pb^2+^ was attracted to the negatively charged carboxyl group of AuNPs-GSH, leading to its attachment to AuNPs/GSH.

Figure 3e displays the XPS of the sensor obtained before and after Pb^2+^ detection. The upper part of Figure 3e shows peaks corresponding to N1s and S2p, suggesting the successful modification of GSH molecules on the surface of the fiber. The lower part of Figure 3e and Figure S7 reveal the emergence of a new Pb4f peak, indicating the successful capture of Pb^2+^ by the GSH molecule on the sensor in the solution.

### 4.2. RI Sensitivity of Sensor

**Figure 4:**
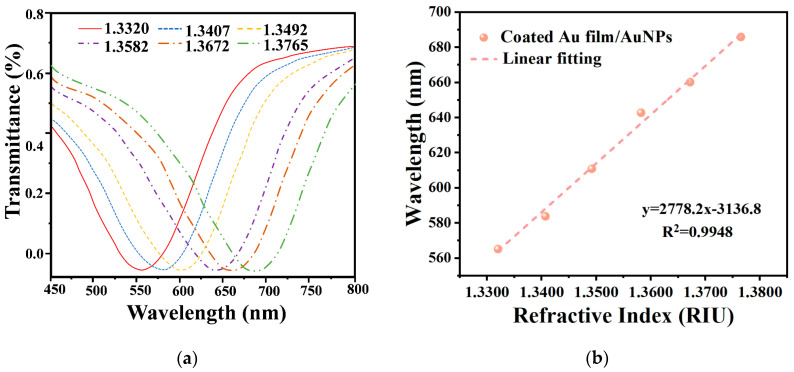
RI sensitivity measurement after the modification of AuNPs. (**a**) Transmission spectrum of SPR fiber modified with AuNPs in NaCl solutions with different RI. (**b**) The linear fitting curve of SPR optical fiber sensors with modified AuNPs.

To further validate the enhanced RI sensitivity brought about by AuNPs, the RI sensitivities of the sensors were measured and recorded before and after modifying the surfaces of the Au film-coated fibers with AuNPs. Figure S8 shows the results of the RI sensitivity tests conducted on the optical fiber sensor coated only with Au film, while Figure 4 depicts the RI sensitivity test results of the optical fiber sensor after modifying the surface of the Au film-coated fiber with AuNPs. The RIs of the surroundings around the sensors were determined using NaCl solutions at concentrations of 0%, 5%, 10%, 15%, 20%, and 25%, corresponding to the RIs of 1.3320, 1.3407, 1.3492, 1.3582, 1.3672, and 1.3765. The transmission spectra of both sensors showed significant redshifts as the RI increased, as shown in Figure 4 and Figure S8. However, there was a notable difference in the total wavelength drifts between the two sensors, with the former experiencing a total wavelength shift of 120.69 nm and the latter experiencing a shift of 98.33 nm. As a result, the RI sensitivities of the two sensors were significantly different, with the former having an RI sensitivity of 2778.2 nm/RIU and the latter possessing an RI sensitivity of 2053.2 nm/RIU. The optical fiber sensor modified with AuNPs demonstrated an approximately 35% higher RI sensitivity compared to the sensor coated only with pure Au film.

### 4.3. Detection of Pb^2+^

**Figure 5:**
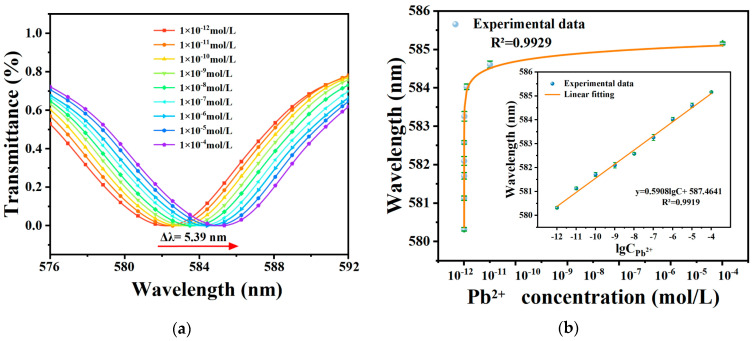
(**a**) Spectral shifts for one dip of the sensor at different concentrations. (**b**) Linear depen-ence of the dip wavelength on the concentration at room temperature.

To evaluate the sensor performance for Pb^2+^ detection, three SPR sensors were employed: one pure Au-coated sensor without AuNPs and GSH modification, one pure Au-coated sensor with GSH modification but without AuNPs, and one Au-coated sensor with both AuNPs and GSH modification. These sensors were used to measure various Pb^2+^ concentrations. The degree of sensor response to Pb^2+^ can be visualized by the magnitude of wavelength shift in the transmission spectra. Figure 5a shows that the dip in the transmission spectrum experiences a notable redshift as the Pb^2+^ concentration increases within the concentration range of 10^−12^ M to 10^−4^ M. The maximum wavelength shift is 5.39 nm, indicating a strong sensor response to low Pb^2+^ concentration (0.43 pM). This sensitivity can be attributed to the formation of chelates between lead ions and GSH, which induces structural changes in the GSH molecule, leading to alterations in light scattering and absorption properties. Consequently, the local RI on the fiber surface undergoes significant changes, resulting in the highly sensitive detection of Pb^2+^. Moreover, the modification of AuNPs induces the LSPR effect on the fiber surface, which further enhances the SPR effect and improves the sensitivity of the optical fiber sensor for Pb^2+^ detection.

A mathematical function was fitted to correlate the wavelength of the transmission spectrum with the Pb^2+^ concentration, and the results are presented in Figure 5b, demonstrating a good linear correlation between the logarithm of Pb^2+^ concentration and the corresponding wavelength. The fitting function, y = 0.5908lgC + 587.4641, yielded an R^2^ value of 0.9919. The sensor demonstrated a sensitivity of 2.32 × 10^11^ nm/(mol/L) for Pb^2+^ detection, and the LOD of the sensor can be estimated to be $C_{LOD}=\delta \lambda /S$ = 0.43 pM [47]. This LOD is significantly below the maximum permissible concentration of Pb^2+^ in drinking water set by the WHO at 10 ppb. However, the fiber optic SPR sensor without AuNPs modification displayed reduced detection sensitivity, higher LOD, and a narrower detection range compared to the sensor modified with AuNPs/GSH. The sensitivity, LOD, and detection range of the unmodified sensor were 2.32 × 10^11^ nm/(mol/L), 0.43 pM, and 10^−12^ M to 10^−4^ M, respectively. Additionally, the optical fiber sensor with unmodified AuNPs and GSH exhibited minimal or negligible response, as detailed in Figure S9.

### 4.4. Stability of the Sensor

**Figure 6:**
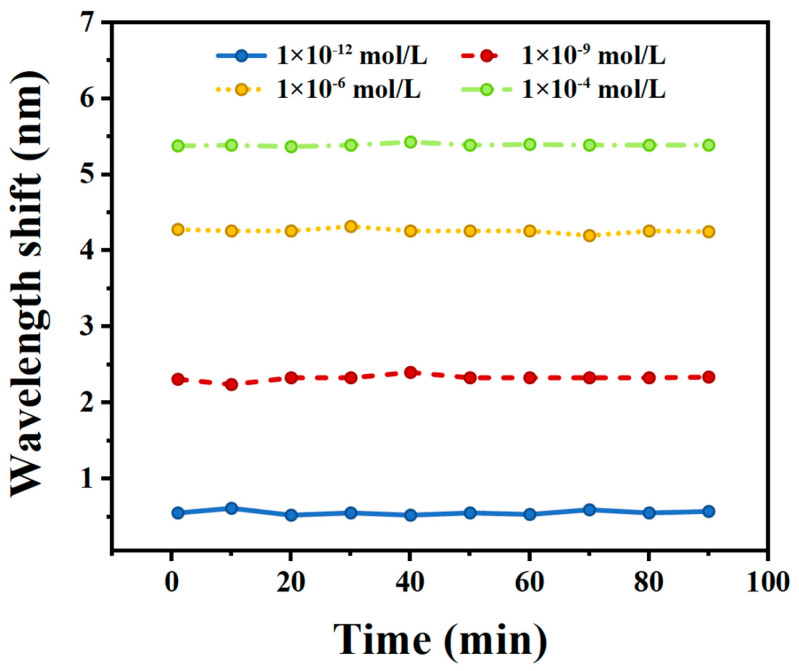
Measurement of the stability of the sensor in three lead ion solutions.

The long-term stability of the ion sensor was measured as well. The sensor was immersed in four Pb^2+^ solutions with concentrations of 10^−12^ M, 10^−9^ M, 10^−6^ M, and 10^−4^ M for a continuous duration of 90 min, and measurements were taken at 10 min intervals. Figure 6 illustrates the measurement results, showing that the maximum deviation of the optical fiber sensor from the initial wavelength is only 0.09 nm, 0.09 nm, 0.07 nm, and 0.03 nm for the four respective concentrations, all falling within the acceptable range of error. These variations may be attributed to background noise from the spectrometer and thus result in minor fluctuations during the experiment. In conclusion, the sensor exhibits excellent stability.

### 4.5. Repeatability of the Sensor

**Figure 7:**
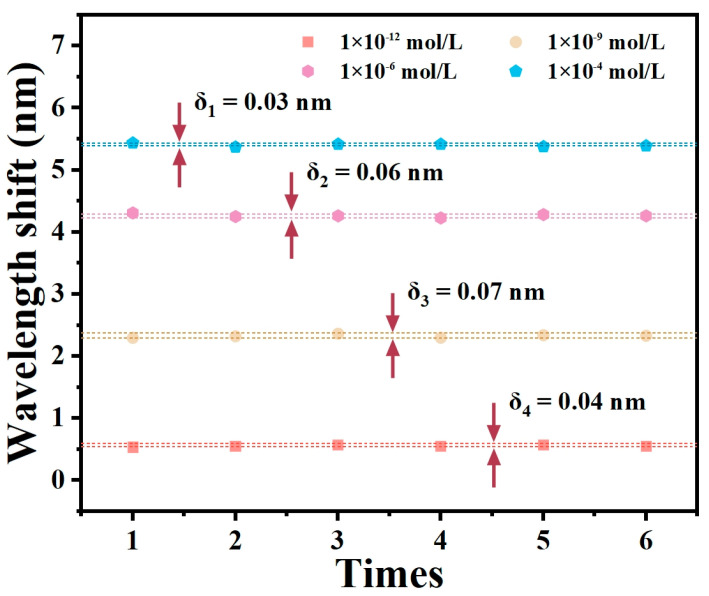
Repeatability of the proposed sensor.

Repeatability is a crucial parameter for evaluating sensor performance. Six repetitions of the test were conducted using Pb^2+^ solutions at concentrations of 10^−12^, 10^−9^, 10^−6^, and 10^−4^ M. The tests were performed on three identical optical fiber SPR sensors that had been modified with AuNPs/GSH. The sensors were cleaned with DI water and alcohol three times after each test and then dried in air to restore their initial state. Figure 7 presents the results of the repeated tests performed on one identical sensor using Pb^2+^ solutions at concentrations of 10^−12^, 10^−9^, 10^−6^, and 10^−4^ M. The wavelength changes exhibited deviations of 0.03, 0.06, 0.07, and 0.04 nm, correspondingly (the other two experiments are shown in Figure S10). These results indicate the consistent repeatability of the optical fiber sensor in detecting Pb^2+^ under various measurements of the same concentration.

### 4.6. Specificity of the Sensor

**Figure 8:**
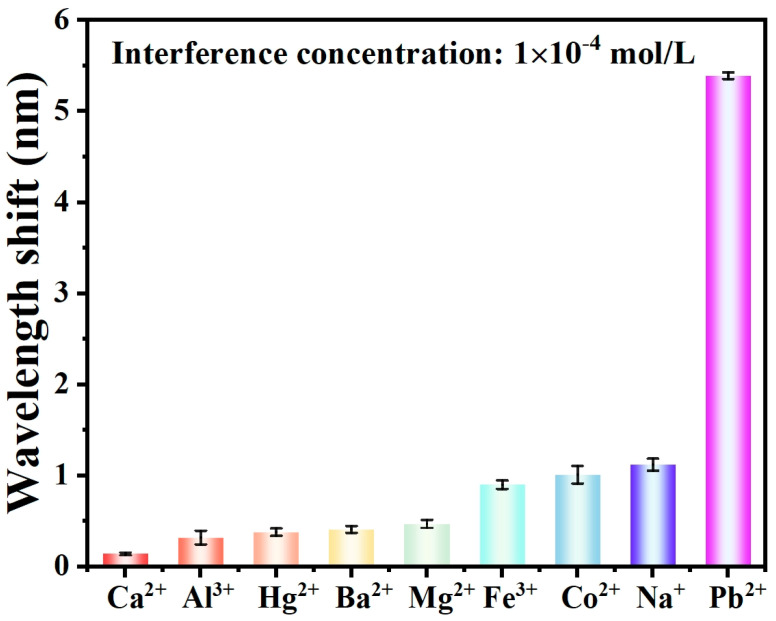
The specificity tests of the sensor for different metal ions.

The specificity of the sensor refers to its capability to selectively recognize specific ions, which is crucial for achieving highly sensitive and accurate measurements. To assess the specificity of the AuNPs/GSH-modified optical fiber sensor for Pb^2+^ detection, the potential interference from other common metal ions (Ca^2+^, Al^3+^, Hg^2+^, Ba^2+^, Mg^2+^, Fe^3+^, Co^2+^, Na^+^) was measured. The same experimental conditions as those used for Pb^2+^ detection were employed, and the concentrations of all metal ions were set to 10^−4^ M (the concentrations of all metal ions at 10^−10^, 10^−9^, 10^−8^, 10^−7^, 10^−6^, and 10^−5^ M are shown in Figure S11). Figure 8 illustrates the wavelength shift of the sensor in various metal ion solutions, indicating that the sensor exhibits minimal response to all metal ions except Pb^2+^. This suggests that the sensor exhibits excellent specificity, which may be attributed to the fact that when the carboxyl group in the GSH molecule forms a ligand bond with Pb^2+^, the carboxyl group (–COOH) undergoes deprotonation while the amino (–NH_2_) group undergoes protonation, and the protonated amino group prevents the binding of GSH to other metal ions, which leads to the selective binding of GSH to Pb^2+^.

### 4.7. Discussions

Here, we summarize the recent research work on Pb^2+^ detection through optical sensors. Their performances were also compared in detail in Table 1. Our sensor exhibits an applicable detection range that surpasses most existing sensors, giving it a clear advantage. Therefore, the sensor could satisfy most requirements for Pb^2+^ measurement. Furthermore, the optical fiber SPR sensor proposed in our work is at a significant advantage in terms of both Pb^2+^ detection sensitivity and the LOD. The sensitivity of our sensor is two orders of magnitude higher than the previously reported highest sensitivity (2.55 × 10^9^ nm/M). Finally, the highly biocompatible material (GSH) we employed as the sensing element holds promise for the development of a novel wearable sensor that enables real-time monitoring of lead ion concentration levels in the human body, which might be achieved by combining a flexible biocompatible plastic optical fiber with the sensitive materials mentioned in this work. Of course, the detection performance of the sensors is yet to be verified in various complex environments for practical applications, but these highly sensitive sensors with universal sensing platforms can be utilized to detect various low concentrations of ions and biochemical molecules by functionalizing the SPR fiber with probes with specificity.

## 5. Conclusions

In summary, a synergistic SPR-enhanced optical fiber sensor functionalized with the self-assembly GSH is developed for highly sensitive and selective detection of lead ions. By modifying AuNPs on the Au film-coated optical fiber, both the RI sensitivity and the specific surface area of the optical fiber improved. This enhancement increases the number of surface active sites and the number of modified probes, greatly improving the sensitivity of the sensor for detecting Pb^2+^. In addition, the presence of deprotonated carboxyl groups in GSH enables stable chelation between Pb^2+^ and GSH, leading to a significant modification in the localized RI on the sensor’s surface, and improves the specificity and long-term stability of Pb^2+^ ion detection. The experimental results demonstrated that the proposed sensor exhibited a significant response across the dynamic concentration range between 10^−12^ and 10^−4^ mol/L, with a high sensitivity of 2.32 × 10^11^ nm/(mol/L). The LOD was determined to be 0.43 pM. Consequently, the optical fiber sensor developed in this study holds great promise for trace detection of Pb^2+^ in various environments, particularly its potential application in the detection of lead contamination in water, food, and biological samples.

## Figures and Tables

**Table 1:** Performance comparison of different optical Pb^2+^ sensors.

Structure/ Method	LOD (pM)	Sensitivity (nm/M)	Dynamic Range (pM)	Sensing Layer	Ref.
Fiber Bragg grating	1.80 × 10^3^	/	1.80 × 10^3^–1.80 × 10^4^	L-glutathione	[19]
Fiber Bragg grating	500.00	2.55 × 10^9^	500–1 × 10^9^	CCS-NGO/PAA nanocomposite	[20]
Fiber Bragg grating	8.56	/	10–10^6^	DNAzyme/AuNPs	[21]
Fiber Bragg grating	9.65 × 10^4^	2.10 × 10^6^	4.49 × 10^5^–4.63 × 10^8^	PMO/BTESPTS	[22]
Tapered fiber	102.47	/	360–1.80 × 10^8^	Black phosphorus	[26]
Tapered fiber	8.60	/	8.60–3019	Black phosphorus	[27]
Tapered fiber	62.20	1.23 × 10^7^	3.02 × 10^2^–3.02 × 10^8^	Black phosphorus	[28]
Interferometric fiber	3019.00	1.03 × 10^7^	3019–1.51 × 10^5^	Chitosan-PVA/ GSH/AuNPs	[30]
Interferometric fiber	2.45 × 10^8^	8.16 × 10^5^	2 × 10^5^–1.20 × 10^6^	Hydroxyethyl methacrylate crosslinked hydrogel	[31]
End reflection fiber	8.00 × 10^8^	280.00	8 × 10^8^–1 × 10^10^	AuNPs/1,1-Mercaptoundecanoic acid	[32]
Plastic clad silica optical fiber	158.00	2.10 × 10^9^	3.60 × 10^4^–7.20 × 10^5^	Pyrrole/CS/ITO/Ag	[48]
ITO glass	50.00	/	100–1 × 10^7^	AuNPs/GSH	[49]
Colorimetry	9.60 × 10^4^	/	9.60 × 10^4^–4.80 × 10^6^	Paper-based/AgNPs/PVA	[50]
Colorimetry	3100.00	5.90 × 10^7^	2.40 × 10^3^–4.80 × 10^4^	AgNPs/dithizone	[51]
Optical sensor	/	2.10 × 10^9^	/	AuNPs/kappa-carrageenan	[52]
Optical sensor	53.00	/	53–2.40 × 10^4^	AuNIs/Poly (m-phenylenediamine-co-aniline-2-sulfonic acid) copolymer nanoparticles	[53]
Optical sensor	720.00	/	/	AuNIs/Poly (m-phenylenediamine-co-Aniline-2- sulfonic acids) copolymer	[54]
LSPR sensor	1.40 × 10^5^	/	1.40 × 10^5^–1.40 × 10^7^	AuNPs/GO/PANI	[55]
Multimode-singlemode-multimode fiber	0.43	2.32 × 10^11^	1–1 × 10^8^	AuNPs/GSH	This Work

## Data Availability

The data presented in this study are available on request from the corresponding author. The data are not publicly available due to privacy restrictions.

## Supplementary Materials

The following supporting information can be downloaded at: https://www.mdpi.com/article/10.3390/ma17010098/s1, Figure S1: The mechanism of GSH-specific capturing Pb^2+^; Figure S2: Measured normalized spectra of MMF-SMF-MMF structures with different sensing lengths: (a) 5mm, (b) 10mm, (c) 15mm, (d) 20mm, (e) 25mm. (f) Plots of surface plasmon resonace peak wavelength versus refractive index for sensors with different sensing lengths; Table S1: Wavelength values at the resonance valley of sensors with different sensing lengths at different refractive index values; Figure S3: Transmittance spectra of sensor of different sensing lengths at n = 1.3320; Table S2: Parameters of gold-plated fiber SPR sensors with different sensing lengths at refractive index value of 1.3320; Figure S4: The preparation of AuNPs; Figure S5: The SPR wavelength variation of the hetero-core fiber coated with Au film, Au film/AuNPs, and Au film/AuNPs/GSH; Figure S6: The elemental distribution on the optical fiber sensor after capturing the Pb^2+^; Figure S7: High-resolution Pb4f spectrum of AuNPs/GSH-modified SPR optical fiber after the addition of Pb^2+^; Figure S8: RI sensitivity measurement of the sensor coated with pure Au-film. (a) Transmission spectra of the sensor coated with pure Au-film in NaCl solutions with different RI. (b) The linear fitting curve of the sensor coated with pure Au-film; Figure S9: Variations in wavelength of the sensors at different Pb^2+^ concentrations; Figure S10: (a) No.1 of repeatability of the proposed sensor. (b) No.2 of repeatability of the proposed sensor; Figure S11: The specificity tests of the sensor for different metal ions at different concentrations. (a) At 1 × 10^−5^ mol/L. (b) At 1 × 10^−6^ mol/L. (c) At 1 × 10^−7^ mol/L. (d) At 1 × 10^−8^ mol/L. (e) At 1 × 10^−9^ mol/L. (f) At 1 × 10^−10^ mol/L.
