# Supplementary material for: Advanced Integration of Glutathione-Functionalized Optical Fiber SPR Sensor for Ultra-Sensitive Detection of Lead Ions
Source: Materials (Basel). 2023 Dec 24;17(1):98. doi: 10.3390/ma17010098 (PMC10780099; doi:10.3390/ma17010098)
Supplement: Supplementary file 1 [file materials-17-00098-s001.zip › materials-2779638-supplementary.pdf]

materials

MDPI

Article

Advanced Integration of Glutathione-Functionalized Optical Fiber SPR Sensor for Ultra-Sensitive Detection of Lead Ions

Jiale Wang [1,†], Kunpeng Niu [1,†], Jianguo Hou [1], Ziyang Zhuang [1], Jiayi Zhu [1], Xinyue Jing [1], Ning Wang [1,*], Binyun Xia [1,*] and Lei Lei [2]

Supplementary Materials

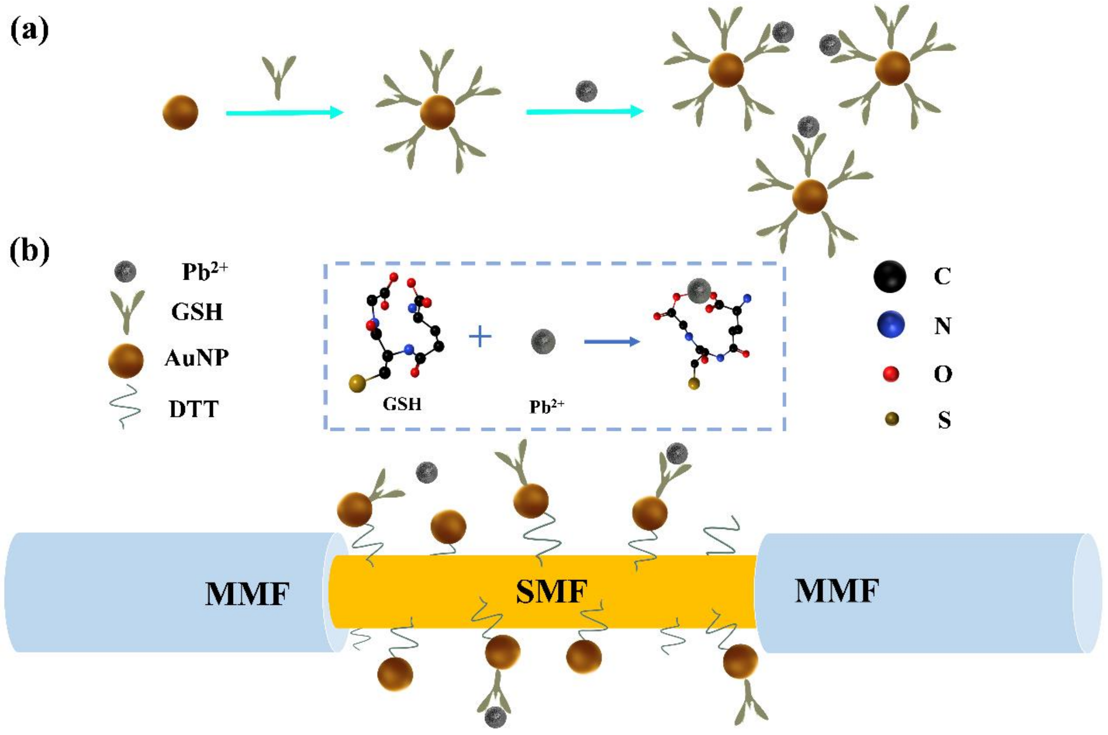

Figure S1. The mechanism of GSH-specific capturing Pb^{2+}.

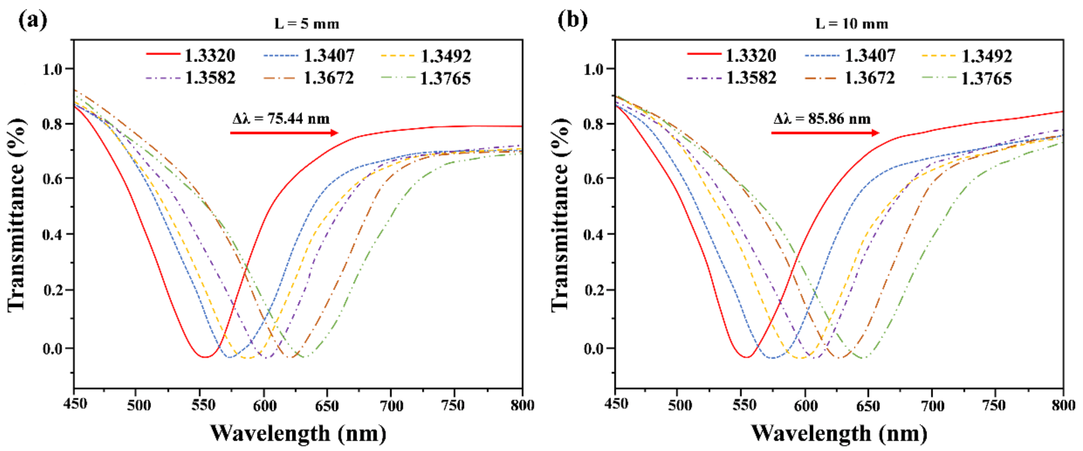

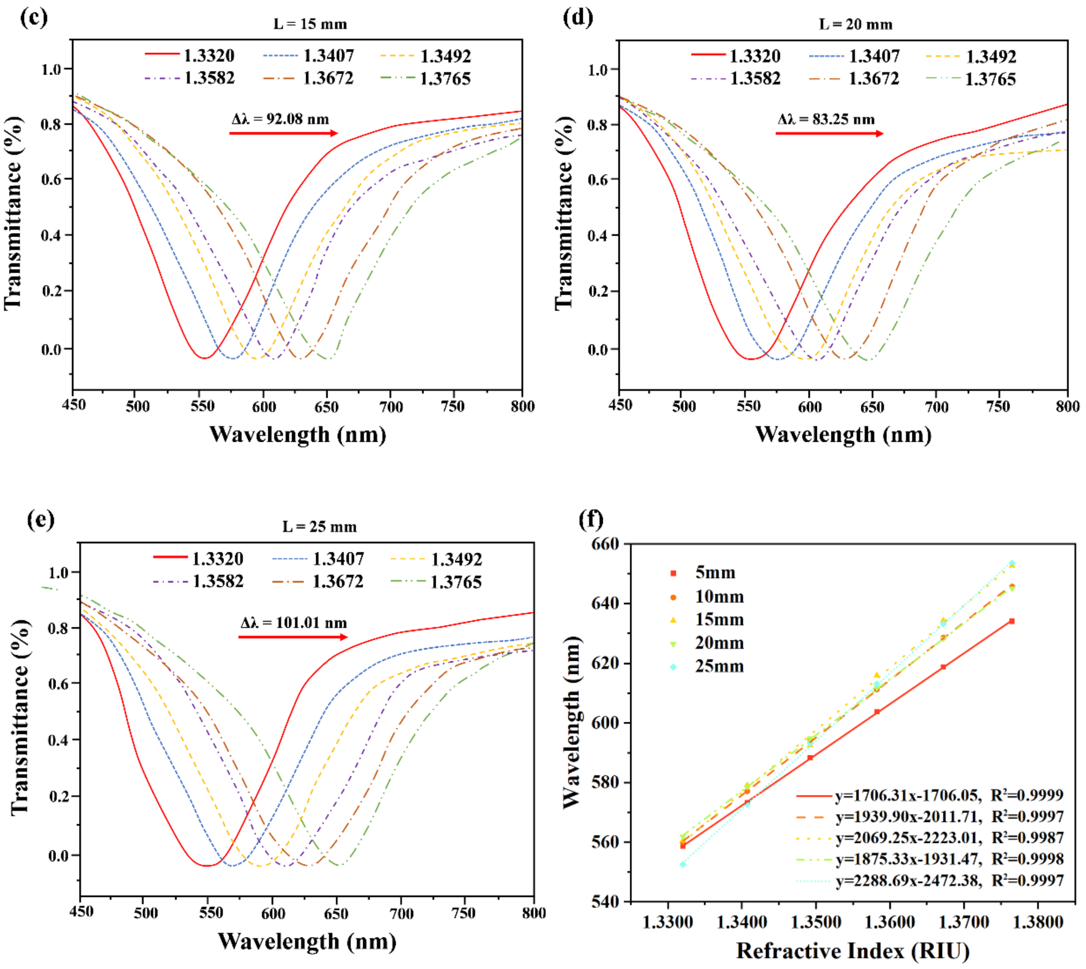

Figure S2. Measured normalized spectra of MMF-SMF-MMF structures with different sensing lengths: (**a**) 5mm, (**b**) 10mm, (**c**) 15mm, (**d**) 20mm, (**e**) 25mm. (**f**) Plots of surface plasmon resonance peak wavelength versus refractive index for sensors with different sensing lengths.

Table S1. Wavelength values at the resonance valley of sensors with different sensing lengths at different refractive index values.

Refractive index (RIU)	Resonance wavelength (5mm)	Resonance wavelength (10mm)	Resonance wavelength (15mm)	Resonance wavelength (20mm)	Resonance wavelength (25mm)
1.3320	558.71	559.93	560.74	561.94	552.62
1.3407	573.39	577.20	579.16	578.63	572.75
1.3492	588.41	594.34	592.57	594.96	593.21
1.3582	603.76	611.33	616.01	611.84	613.14
1.3672	618.84	628.67	634.41	628.38	633.29
1.3765	634.15	645.79	652.82	645.19	653.63

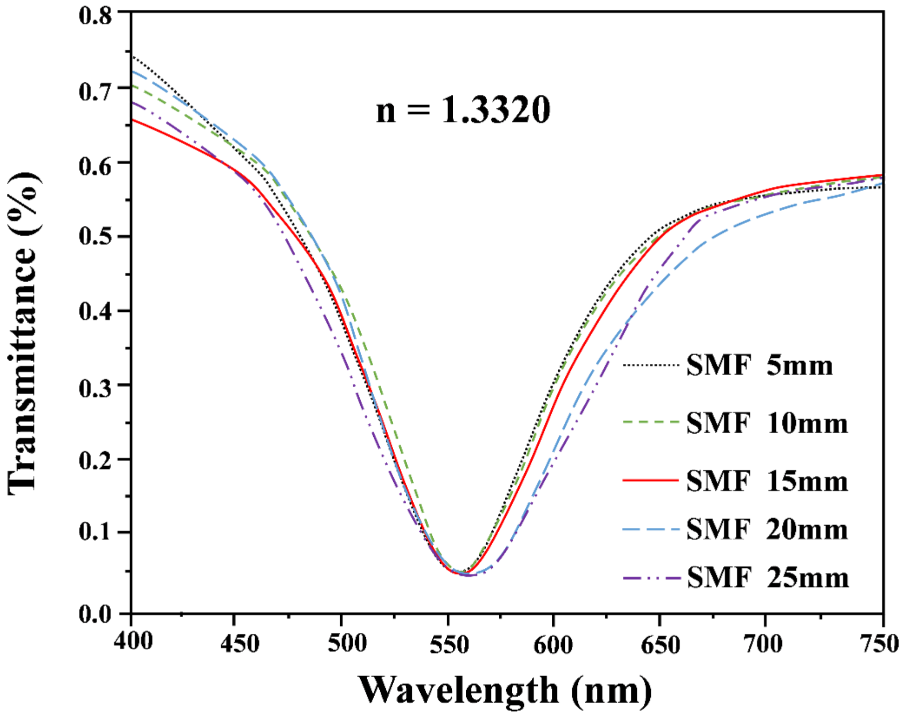

Figure S3. Transmittance spectra of sensor of different sensing lengths at n=1.3320.

Table S2. Parameters of gold-plated fiber SPR sensors with different sensing lengths at refractive index value of 1.3320.

L(mm)	S_n (nm/RIU)	FWHM (nm)	FOM
5	1706.31	65.62	26
10	1939.90	62.58	31
15	2069.25	66.75	31
20	1875.33	78.24	24
25	2288.69	108.99	21

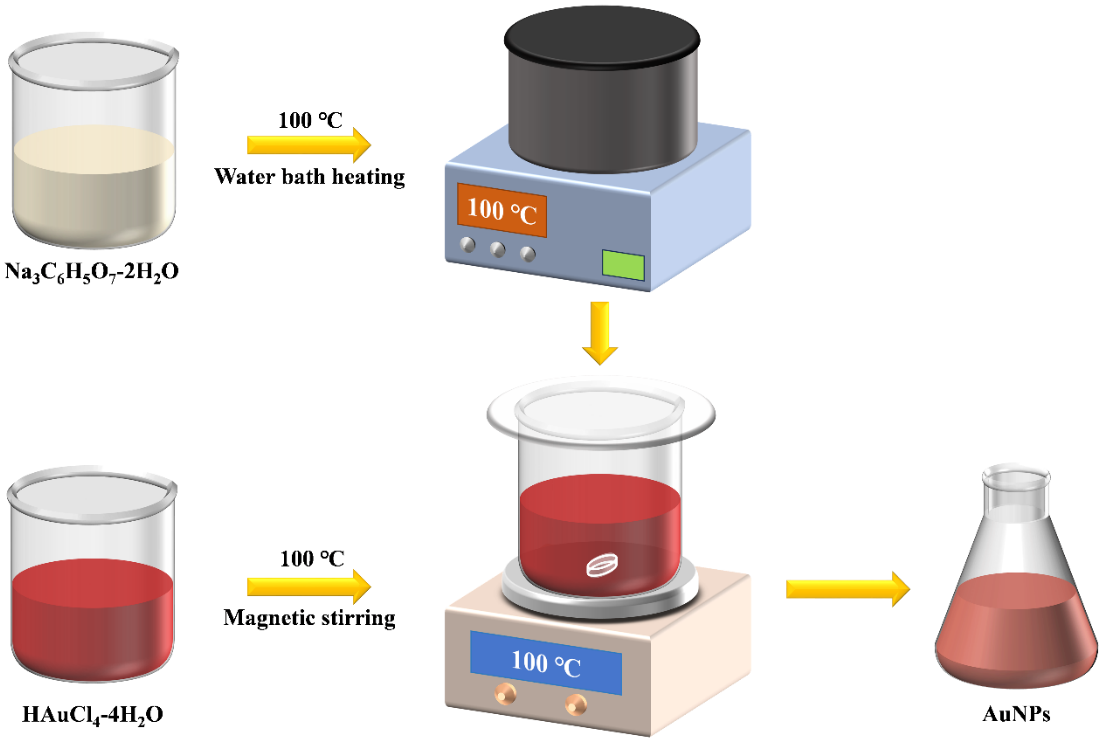

Figure S4. The preparation of AuNPs.

The preparation process of AuNPs is shown in Fig. S1. A solution of 2.9×10^{-4} M chloroauric acid tetrahydrate ($HAuCl_4$-$4H_2O$) and a solution of 3.88×10^{-2} M sodium citrate ($Na_3C_6H_5O_7$-$2H_2O$) were prepared in deionized water. The sodium citrate solution was heated to 100°C in a water bath and then left to stand. Next, a 50 ml $HAuCl_4$-$4H_2O$ (2.9×10^{-4} M) solution was sealed with plastic wrap, vigorously stirred on a thermostatic magnetic stirrer, and heated to 100°C. Then, 3 ml of $Na_3C_6H_5O_7$-$2H_2O$ (3.88×10^{-2} M) solution was added, and stirring was continued for 8 min. The solution changed to burgundy color, indicating the successful synthesis of AuNPs (20 nm in diameter).

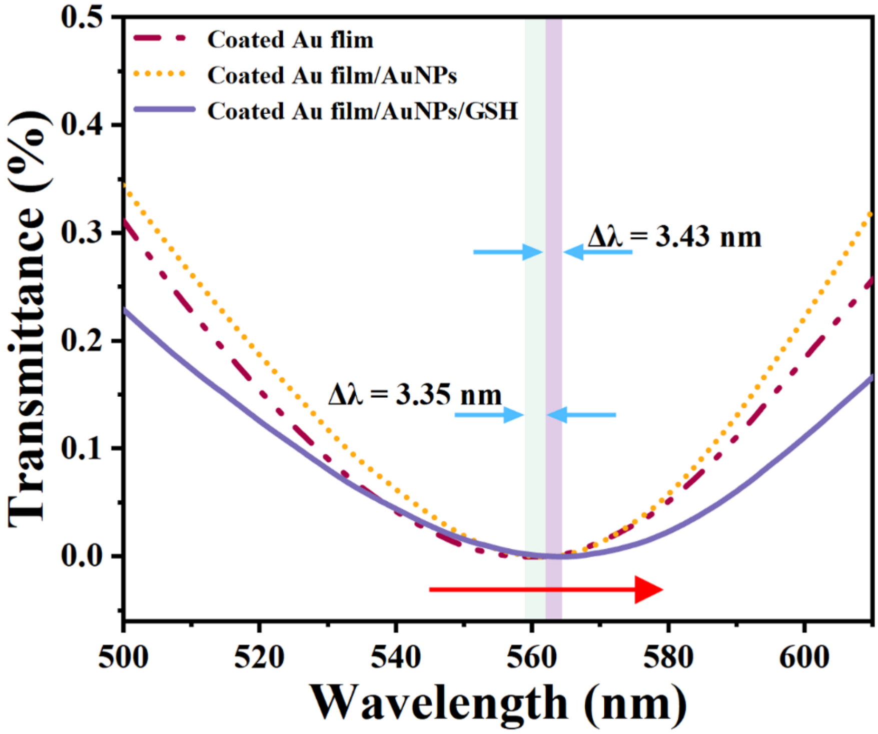

Figure S5. The SPR wavelength variation of the hetero-core fiber coated with Au film, Au film/AuNPs, and Au film/AuNPs/GSH.

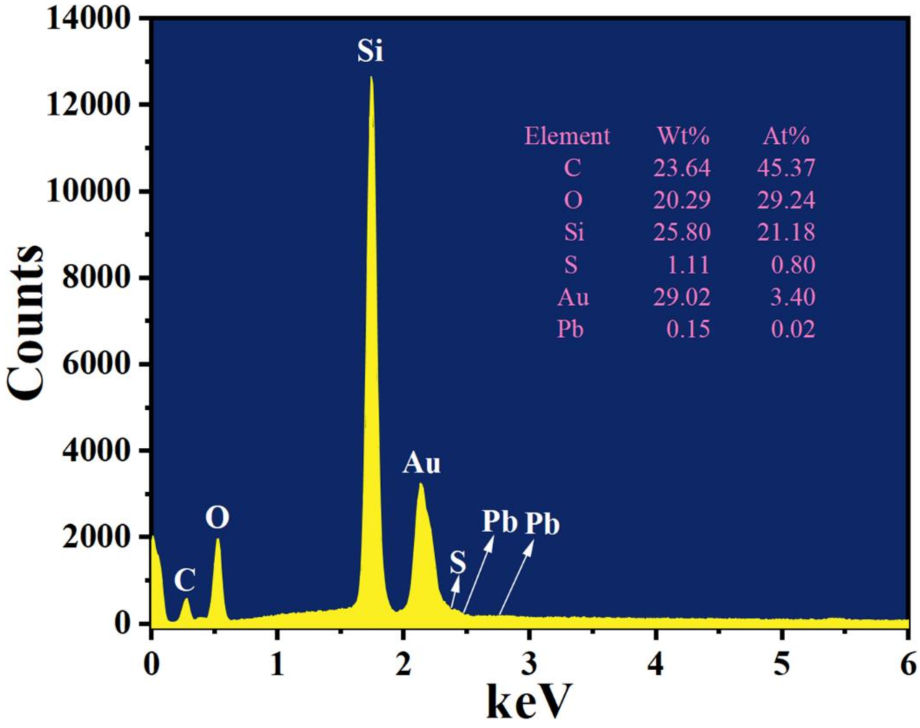

Figure S6. The elemental distribution on the optical fiber sensor after capturing the Pb^{2+}.

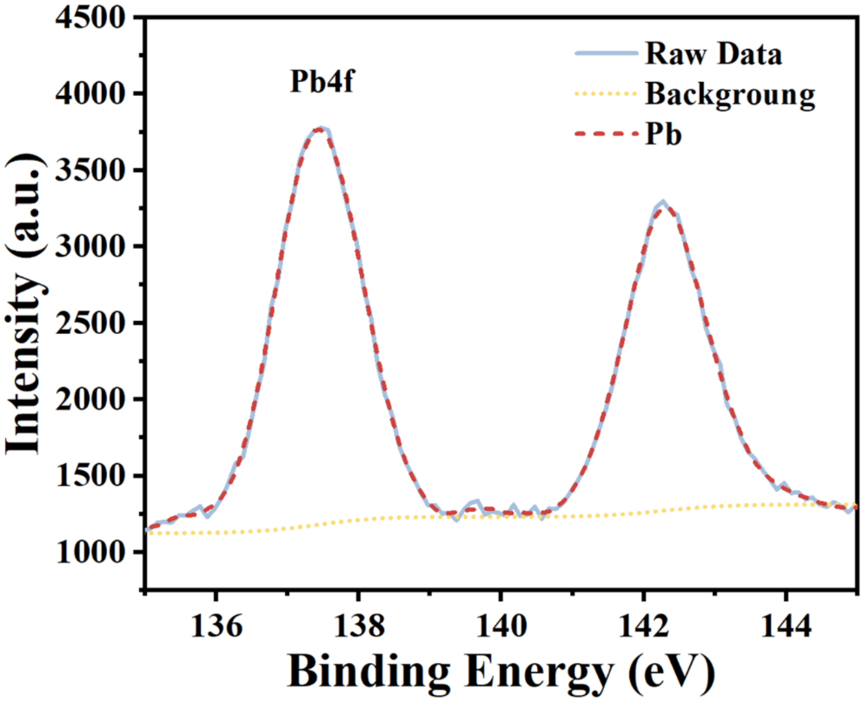

Figure S7. High-resolution Pb4f spectrum of AuNPs/GSH-modified SPR optical fiber after the addition of Pb^{2+}.

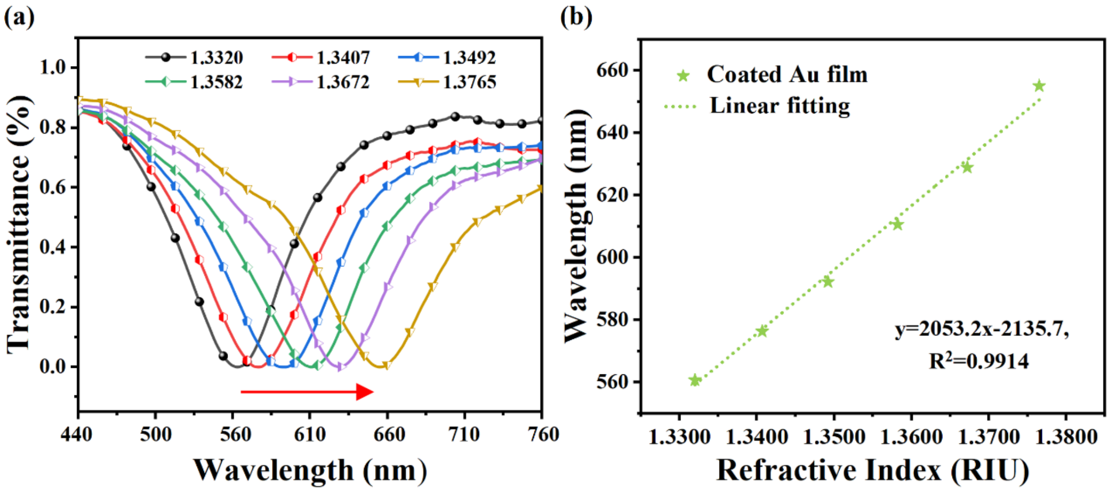

Figure S8. RI sensitivity measurement of the sensor coated with pure Au-film. (**a**) Transmission spectra of the sensor coated with pure Au-film in NaCl solutions with different RI. (**b**) The linear fitting curve of the sensor coated with pure Au-film.

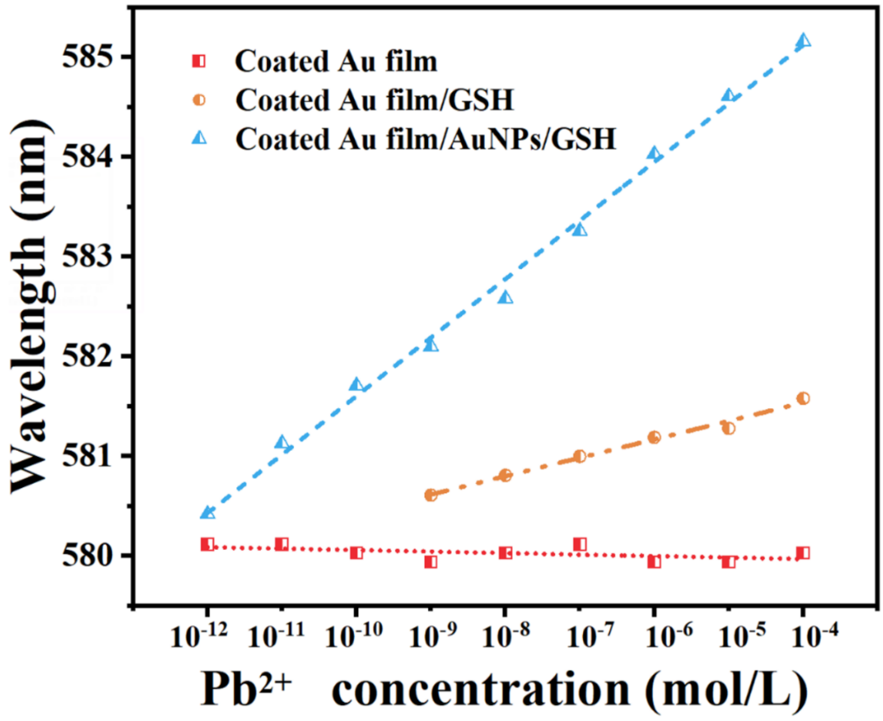

Figure S9. Variations in wavelength of the sensors at different Pb^{2+} concentrations.

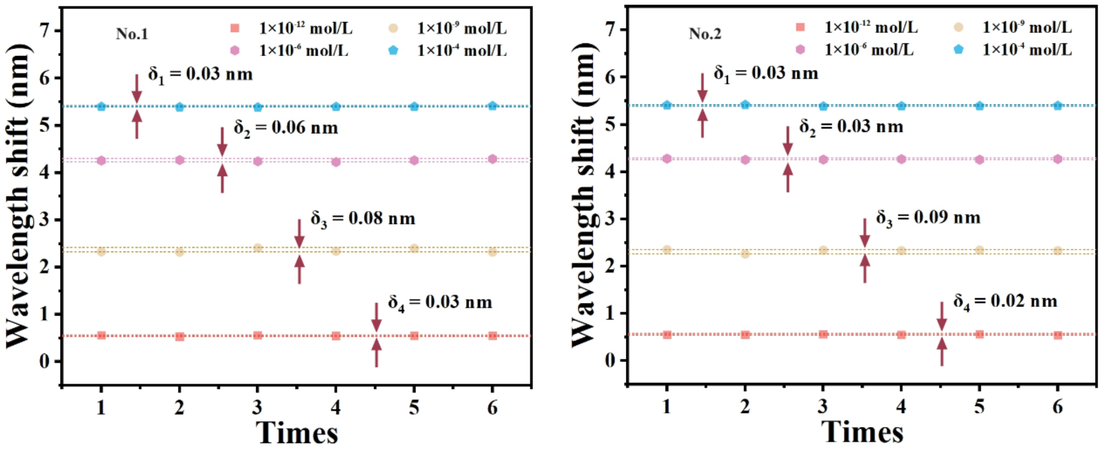

(**a**) (**b**)

Figure S10. (**a**) No.1 of repeatability of the proposed sensor. (**b**) No.2 of repeatability of the proposed sensor.

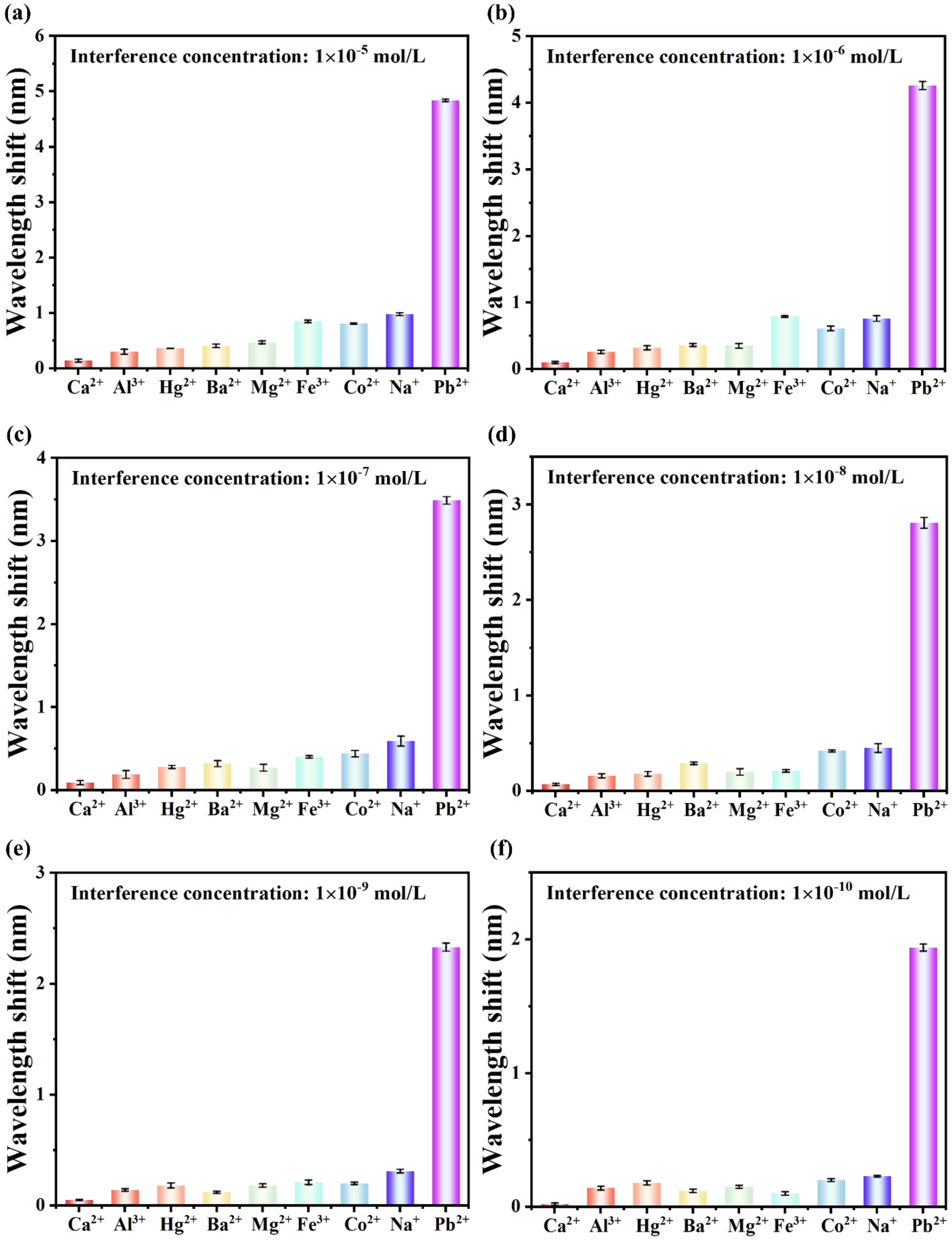

Figure S11. The specificity tests of the sensor for different metal ions at different concentrations. (**a**) At 1 × 10^{-5} mol/L. (**b**) At 1 × 10^{-6} mol/L. (**c**) At 1 × 10^{-7} mol/L.(**d**) At 1 × 10^{-8} mol/L. (**e**) At 1 × 10^{-9} mol/L. (**f**) At 1 × 10^{-10} mol/L.
